# New Lipophilic Piceatannol Derivatives Exhibiting Antioxidant Activity Prepared by Aromatic Hydroxylation with 2-Iodoxybenzoic Acid (IBX)

**DOI:** 10.3390/molecules14114669

**Published:** 2009-11-17

**Authors:** Roberta Bernini, Maurizio Barontini, Carmela Spatafora

**Affiliations:** 1 Agrobiology and Agrochemistry Department, University of Tuscia, Via S. Camillo De Lellis, 01100 Viterbo, Italy; 2 Department of Chemical Sciences, University of Catania, Via A. Doria 6, I-95125 Catania, Italy; E-Mail: cspatafo@unict.it (C.S.)

**Keywords:** aromatic hydroxylation, 2-iodoxybenzoic acid (IBX), lipophilic piceatannol derivatives, lipophilic antioxidants

## Abstract

Piceatannol (*E*-3,5,3’,4’-tetrahydroxystilbene) is a phytoalexin synthesized in grapes in response to stress conditions. It exhibits strong antioxidant and antileukaemic activities due to the presence of the catechol moiety. To modify some physical properties like solubility, and miscibility in non-aqueous media some new previously unreported piceatannol derivatives having lipophilic chains on the A-ring were prepared in good yields by a simple and efficient procedure. The key step was a chemo- and regioselective aromatic hydroxylation with 2-iodoxybenzoic acid (IBX). The new compounds showed antioxidant activity and seemed promising for possible applications as multifunctional emulsifiers in food, cosmetic and pharmaceutical fields.

## 1. Introduction

**Figure 1:**
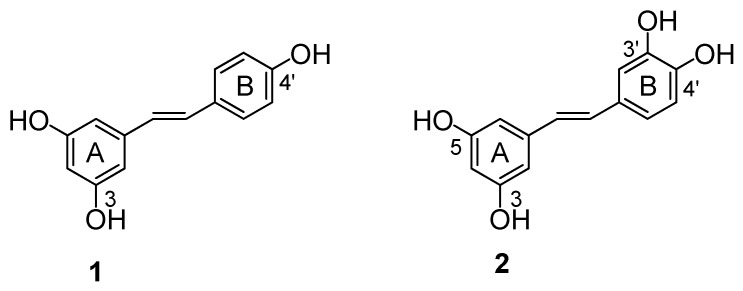
Chemical structure of natural stilbenes **1** and **2**.

In the last few years, stilbene-based compounds have attracted the attention of many researchers due to their wide range of positive biological effects [1,2,3]. One of the most relevant and extensively studied compounds is resveratrol (*E*-3,5,4’-trihydroxystilbene **1**, Figure 1), a phenolic compound found mainly in grapes, where it is synthesized in response to stress conditions such as fungal infection and trauma [4,5]. In red wines it is present at concentrations of mg/L and it is well known that a moderate alcohol consumption has some protective effects against coronary heart disease [6]. Several studies have demonstrated that resveratrol exhibits a wide range of biological and pharmacological activities [7,8]. For example, it has been shown to have cancer preventive properties [9,10,11], anti-inflammatory activities [12], and a role in the prevention of atherosclerosis and coronary diseases [13,14].

Structurally similar to resveratrol is piceatannol (*E*-3,5,3’,4’-tetrahydroxystilbene **2**) which differs from **1** in that it possesses an extra hydroxyl group adjacent to the active 4’-OH of resveratrol. It is present in low quantity in grapes [15], peanuts [16], *Euphorbia lagascae* [17] and *Vaccinium* berries [18] and, like resveratrol, it is a phytoalexin. Piceatannol (**2**) shows many biological activities [19,20,21]. It has known anticancer and antileukaemic properties, inducing apoptosis in several cell lines and animal models; it inhibits a variety of tyrosinase kinases involved in cell proliferation [22,23]. A recent study has demonstrated that the cancer preventative properties of resveratrol (**1**) are related to its natural conversion into metabolite piceatannol (**2**) in living cells by the enzyme CYP1B1 (belongs to the cytochrome P450 enzyme family) that is over-expressed in a wide variety of human tumors [24]. Other experimental evidences showed that piceatannol has a higher level of antioxidant activity compared to resveratrol [25]. This result is according to the evidence that a catechol moiety present in a compound increases the cytotoxic and antioxidant activity *in vitro* [26,27]. 

As all naturally occurring phenolic antioxidants, stilbene derivatives **1** and **2** are hydrophilic and thus scarcely soluble in aprotic solvents, oils and emulsions. Consequently, their application in the food, pharmaceutical and cosmetic fields is strongly limited. 

In recent times, more attention has been turned on the development of possible strategies to modify and to enhance the functional properties of natural antioxidants without altering the functionality responsible for the biological activities. For example, the hydrophobicity of antioxidant phenolic acids has been enhanced by esterifying the carboxylic acid moiety of the phenolic acid with a fatty alcohol by chemical and enzymatic procedures [28]. In a similar way, phenethyl alcohols have been lipophilized by esterifying the alcoholic function with acid chlorides or acid anhydrides [29]. In this way amphiphilic phenolic compounds that are able to distribute between the aqueous phase and the lipophilic phase and interact with an emulsifier at the interface were obtained. Recently, it has been reported that these compounds are potentially interesting for possible applications in nanotechnology [30]. To the best of our knowledge, lipophilic piceatannol derivatives have not been reported in the literature and no chemical or enzymatic synthetic procedures are described. 

**Figure 2:**
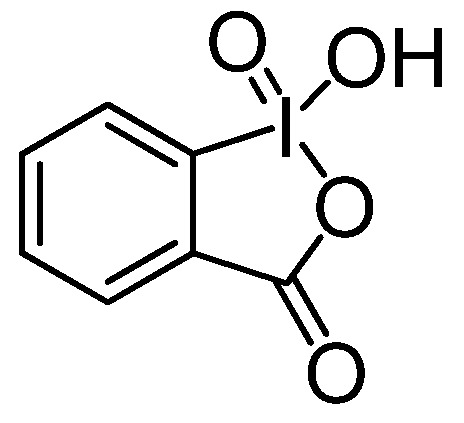
Chemical structure of 2-iodoxybenzoic acid (IBX).

In the last few years, our research has been devoted to the preparation of biologically active catecholic compounds from naturally occurring phenols by oxidative procedures. For example, we obtained in very good yields 2-(3’,4’-dihydroxyphenyl)ethanol (hydroxytyrosol) and its lipophilic derivatives, useful molecules for cosmetic and pharmaceutical applications [31,32,33,34,35]; novel lignans showing antioxidant activity [36]; endogenous catecholamines utilized for the treatment of many clinical disorders, including hypertension and cardiovascular diseases [37]. The oxidant of choice was 1-hydroxy-1-oxo-1*H*-1λ^5^-benzo[d][1,2]iodoxol-3-one (2-iodobenzoic acid, IBX, Figure 2), the most important representative of pentavalent iodine heterocycles first prepared by Hartmann and Mayer [38] which has attracted increasing interest during the last decade because of its selective, mild and environmentally friendly properties as an oxidant in organic synthesis [39,40,41]. Among its synthethic potential, it is particularly interesting the ability to perform a regioselective oxidation of phenols into *ortho*-quinones [42,43,44]. When the oxidative step was followed by *in situ* reduction, a catechol moiety was produced with a selectivity similar to that of cytochrome P450 enzymes [45].

In this paper we describe a simple and efficient route to prepare lipophilic piceatannol derivatives displaying free-catecholic functionality in the B ring and long alkyl chains in the A ring by an oxidative procedure using IBX and subsequent reduction with sodium dithionite (Na_2_S_2_O_4_). As examples of chains with different length, we choose acetyl (C_2_), butanoyl (C_4_), octanoyl (C_8_) and palmitoyl (C_16_). These new compounds were then evaluated for their radical-scavenging activity using the DPPH method [46].

## 2. Results and Discussion

### 2.1. Preparation of 3,5-di-O-acyl resveratrol derivatives ***4a-d***

**Scheme 1:**
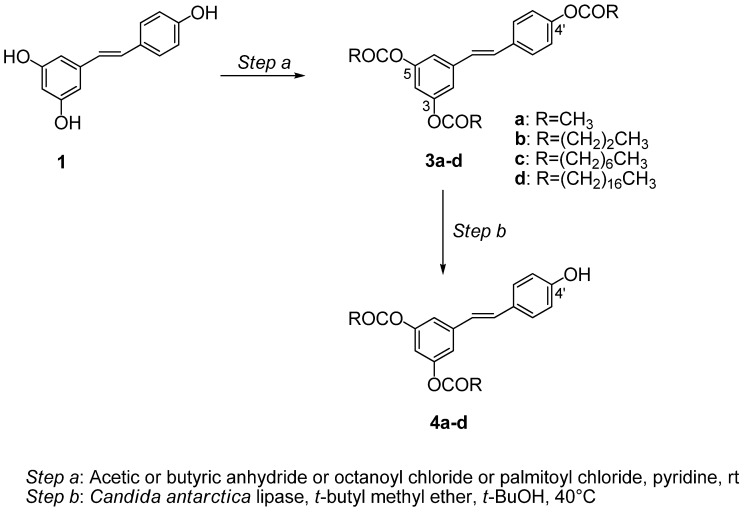
Synthesis of 3,5-di-*O*-acetylresveratrol derivatives **4a-d**.

Firstly, we prepared 3,5-di-*O*-acyl resveratrol derivatives **4a-d** by a two-step procedure (Scheme 1). Peracylated derivatives [3,5,4’-tri-*O*-acetyl resveratrol (**3a**); 3,5,4’-tri-*O*-butanoyl resveratrol (**3b**); 3,5,4’-tri-*O*-octanoyl resveratrol (**3c**) and 3,5,4’-tri-*O*-palmitoyl resveratrol (**3d**)] were obtained in 90–95% yields from resveratrol (**1)** using acetic and butyric anhydride in pyridine and octanoyl and palmitoyl chloride in pyridine, respectively (*step a*); then, compounds **3a-d** were dispersed in *t*-butyl methyl ether and *n*-butanol and subjected to enzymatic alcoholysis with *Candida antarctica* lipase to isolate the corresponding 3,5-di-*O*-acetylated resveratrol derivatives **4a-d** in 60–90% yields (*step b*) [47,48].

### 2.2. Aromatic hydroxylation of compounds ***4a-d***

**Scheme 2:**
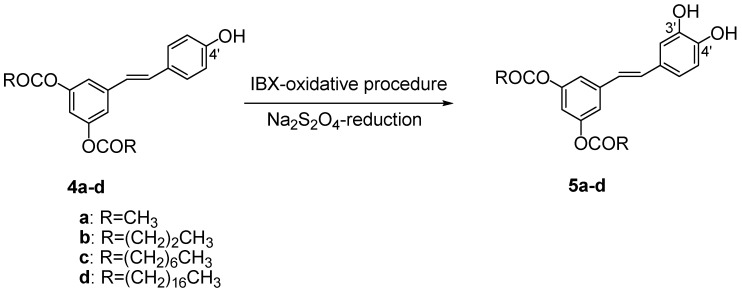
Aromatic hydroxylation of 3,5-di-*O*-acyl resveratrol derivatives **4a-d**.

The key reaction for the preparation of 3,5-di-*O*-acyl piceatannol derivatives **5a-d** was the insertion of an hydroxyl group in C-3’ (B ring) of compounds **4a-d** (Scheme 2). The model substrate for the optimization of the reaction conditions was 3,5-di-*O*-acetyl resveratrol (**4a**).

We performed some experiments varying the solvent, reaction time, and temperature (Table 1). In all cases an excess of IBX was used (1.2 equiv.); Na_2_S_2_O_4_ in tetrahydrofuran (1.0 M) was the reducing solution. Reactions were monitored by chromatographic analysis (TLC and HPLC). On the basis of the literature data [49], first we used methanol as solvent and a low temperature reaction but under these experimental conditions 3,5-di-*O*-acetyl piceatannol (**5a**) was isolated in low yield (Table 1, entry 1). The best result in terms of solubility, conversion and yield was obtained by dissolving compound **4a** in dimethyl sulfoxide (DMSO) at room temperature. In these experimental conditions, piceatannol derivative **5a** was isolated in quantitative yield after only 1 h (Table 1, entry 4). The oxidative step was both chemoselective and regioselective the double bond present of **5a** being unaffected. 

**Figure 4:**
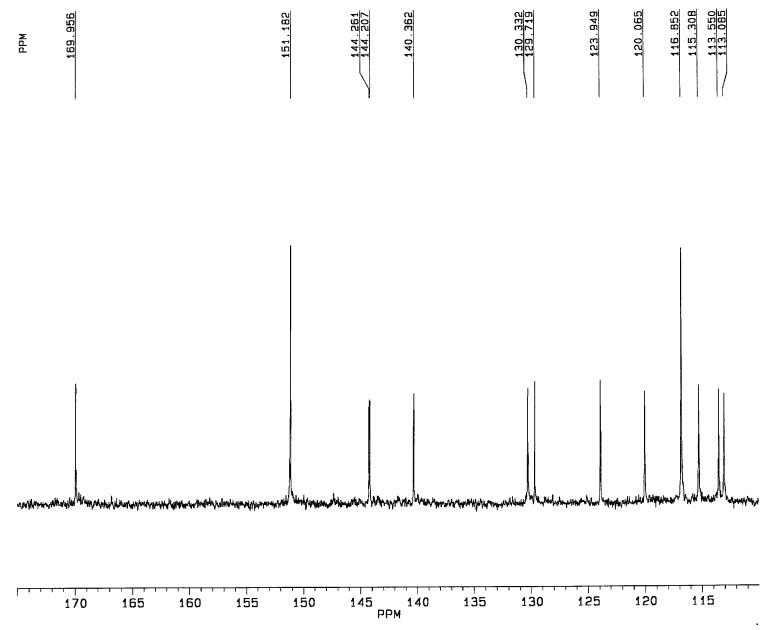
^13^C-NMR spectrum (180-110 ppm region) of 3,5-di-*O*-acetyl piceatannol (**5a**).

A significative region of the ^13^C-NMR spectrum of the new synthesized 3,5-di-*O*-acetyl piceatannol (**5a)** is shown in Figure 4. The presence of the catecholic moiety in the B-ring of 3,5-di-*O*-acetyl piceatannol (**5a**) was confirmed by analyzing this spectroscopic region, which showed the lack of symmetry of the B-ring, two quaternary carbon atoms (C-3’ and C-4’) at 140.4 and 144.2 ppm and three C-H carbons (C-2’, C-5’, C-5’) at 113.5, 115.3 and 116.8 ppm, respectively.

**Scheme 3:**
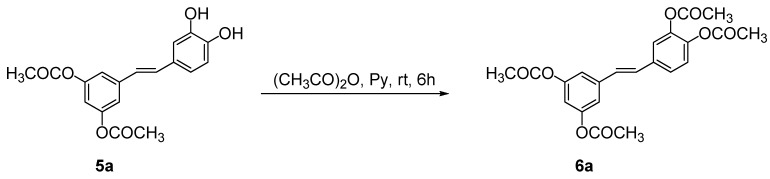
Acetylation of 3,5-di-*O*-acyl piceatannol derivative **4a**.

Furthermore, the presence of two free hydroxyl groups in **5a** was chemically demonstrated by an esterification reaction of **5a** under classical conditions with acetic anhydride in pyridine (Scheme 3). As expected, the proton magnetic resonance spectrum (^1^H-NMR) of the resulting product **6a** evidenced the presence of two singlets at 2.23 and 2.25 ppm attributable to the twelve protons of the four acetyl groups of **6a**. 

As already reported, a plausible ionic mechanism for the regioselective aromatic hydroxylation of phenol derivatives was reported by Pettus *et al*. [42]. The phenol added to the iodine-(V) center of IBX to give a λ^5^-iodanyl intermediate and to extrude H_2_O. The intramolecular (and regioselective) delivery of an oxygen atom from this specie lead to a λ^3^-iodanyl intermediate which undergoes tautomerization and oxidatively collapses to produce an *ortho*-quinone derivative and 2-iodobenzoic acid (IBA), the only by-product of the reaction. The catecholic moiety was finally obtained after the *in situ* reductive step.

On the basis of the good results, this simple efficient and useful synthetic procedure was applied to 3,5,4’-tri-*O*-butanoyl resveratrol (**4b**); 3,5,4’-tri-*O*-octanoyl resveratrol (**4c**) and 3,5,4’-tri-*O*-palmitoyl resveratrol (**4d**) (Scheme 2). The corresponding lipophilic piceatannol derivatives **5b-d** were isolated in satisfactory yields (Table 2, entry 1-3) and with a high degree of purity.

### 2.3. Evaluation of the antioxidant activity of ***5a-d***

**Figure 5:**
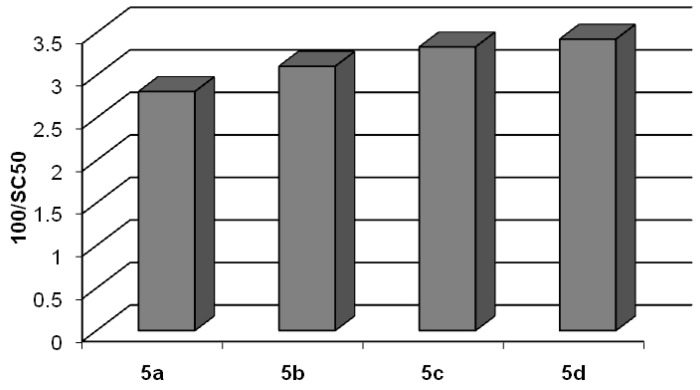
DPPH• scavenging activity of compounds **5a**-**d** expressed as 100/SC_50_.

After their preparation, we evaluated the radical scavenging activity of compounds **5a-d** using the stable 2,2-diphenyl-1-picrylhydrazyl (DPPH•) radical commonly and widely utilized for testing this activity in natural phenolic compounds. Experimental results are reported in Table 3 and Figure 5. The parameter SC_50_ is the Scavenging Capacity that is the concentration of compound (expressed in μM) able to quench the 50% of DPPH radical in a 184 μM solution (see Experimental section for details). As shown, SC_50_ values of lipophilic derivatives **5a-d** were in the range 28.9–35.6 μM. All tested compounds exhibited an antioxidant activity superior to that of resveratrol **1** and comparable to that of piceatannol **2** [50]. The length of the alkyl chain does not influence the radical-scavenging activity. 

## 3. Experimental

### 3.1. Materials and methods

Resveratrol was purchased from Aldrich Company (Milan, Italy) as well all other solvents and reagents. All chemicals used were of analytical grade. IBX was prepared in our laboratory as described in the literature [51]. The *Candida antarctica* lipase (Chirazyme L-2, c. –f., C2, Lyo) was a gift from Roche. HPLC analyses were performed on a Varian Prostar 325 apparatus equipped with a Varian Pursuit 5μ C_18_ column (150 × 4.6 mm) and a dual wavelength UV-Vis detector selected on λ = 280 nm. Elutions were carried out at a 1 mL/min flow rate using a H_2_O/CH_3_CN mixture (90:10, v/v) for the first minute and a gradient to 40:60 within the following 30 minutes. ^1^H- and ^13^C-NMR spectra were recorded in CD_3_COCD_3_ or CDCl_3_/CD_3_OD using a Bruker 200 MHz spectrometer. All chemical shifts are expressed in parts per million (δ scale) and referenced to either the residual protons or carbon of the solvent. HRMS were recorded with Micromass Q-TOF Spectrometer (Waters). Compounds **4a-d** were purified by flash chromatography on silica gel using the Buchi-Biotage FLASH 12™ system equipped with prepacked silica gel cartridges 12S. Analytical Thin-Layer Chromatography (TLC) were performed on silica gel (Merck 60 F_254_) plates using cerium sulphate as developing reagent. 

### 3.2. Preparation of 3,5-di-O-acyl piceatannol derivatives ***5a-d***

*Step a. Acylation reactions of resveratrol* (**1**)**.** Peracylated derivatives [3,5,4’-tri-*O*-acetylresveratrol (**3a**); 3,5,4’-tri-*O*-butanoylresveratrol (**3b**); 3,5,4’-tri-*O*-octanoylresveratrol (**3c**) and 3,5,4’-tri-*O*-palmitoylresveratrol (**3d**)] were prepared using acetic and butyric anhydride in pyridine (1:1) and octanoyl and palmitoyl chloride in pyridine (1:1), respectively. The solutions were stirred at room temperature for 6-12 h; then, they were acidified with HCl 2N and extracted with ethyl acetate; the organic layer was washed with a solution of NaHCO_3_, and dried with Na_2_SO_4_. The solvent was removed under reduced pressure to afford the peracylated derivatives **3a-d** in 90-95% yields. 

*Step b. Enzymatic alcoholysis of 3,5,4’-triO-acylresveratrol derivatives* **3a-d**. Compounds **3a-d** (200 mg) were dispersed in *t*-butyl methyl ether (20 mL) and *n*-butanol (0.8 mL); *Candida antarctica* lipase (200 mg) was added to the solution of **3a-d** and the mixture was shaken (400 rpm) at 40 °C. Finally, reactions were quenched by filtering off the enzyme and the filtrate was evaporated *in vacuo*. The residue was subjected to flash chromatography to obtain the di-*O*-acyl derivatives **4a-d** in 60–90% yields. MS-FAB and ^1^H-NMR spectra of **3a-d** and **4a-d** were identical to those reported in the literature [47,48].

### 3.3. Aromatic hydroxylation of 3,5-di-O-acyl resveratrol derivatives ***4a-d***

Compounds **4a-d** (0.5 mmol) were dissolved in dimethyl solfoxide (5 mL) and then 2-iodoxy-benzoic (IBX) was added (1.2 eq.). The mixture was kept under magnetic stirring at room temperature and monitorated by TLC. When the substrates disappeared, water (5 mL) and sodium dithionite (0.5 mmol) were added. A chromatic change of the solution from red to colorless was observed. After 5 minutes, the mixtures were extracted with ethyl acetate (3 × 10 mL). The organic phases were washed with a solution of NaHCO_3_and then dried on Na_2_SO_4_. The organic solvent was evaporated under reduced pressure obtaining a crude. Final products **5a**-**d** were purified by chromatographic purification on silica gel and characterized by spectroscopic analysis. 

*3,5-Di-O-acetylpiceatannol* (**5a**). Colorless oil, quantitative yield. ^1^H-NMR (CD_3_COCD_3_) δ 2.27 (s, 6H, 2 × C*H*_3_), 2.99 (2H, O*H*), 6.80-7.20 (m, 8H, C*H_α_*=C*H_β_*, *H*-2,4,6,2’,5’,6’); ^13^C-NMR (CD_3_COCD_3_) δ 21.1, 113.1, 113.6, 115.3, 116.9, 120.1, 123.9, 129.7, 130.3, 140.4, 144.2, 144.3, 151.2, 170.0; HRMS found: 328.22; C_18_H_16_O_6_ requires 328.09.

*3,5-Di-O-butanoyl piceatannol* (**5b**). Yellow oil, 88% yield. ^1^H-NMR (CD_3_COCD_3_) δ 1.02 (t, 6H, *J* = 7.3 Hz, 2 × C*H*_3_), 1.65-1.83 (m, 4H, 2 × C*H*_2_CH_3_), 2.56 (t, *J* = 9.8 Hz, 4H, OCOC*H*_2_), 6.79-7.20 (m, 8H, C*H_α_*=C*H_β_*, *H*-2,4,6,2’,5’,6’); ^13^C-NMR (CD_3_COCD_3_) δ 13.7, 18.9, 36.4, 114.0, 114.9, 116.2, 117.3, 120.3, 124.8, 130.1, 131.5, 141.1, 146.1, 146.5, 152.6, 172.1; HRMS found: 384.34; C_22_H_24_O_6_ requires 384.16.

*3,5-Di-O-octanoyl piceatannol* (**5c**). Yellow oil, 84% yield. ^1^H-NMR (CD_3_COCD_3_) δ 0.86 (m, 6H, 2 × C*H*_3_), 1.28-1.42 (m, 16H, 8 × C*H*_2_), 1.62-1.76 (m, 4H, 2 × C*H*_2_), 2.56 (t, *J* = 7.2 Hz, 4H, 2 × C*H*_2_), 6.75-7.15 (m, 8H, C*H_α_*=C*H_β_*, *H*-2,4,6,2’,5’,6’); ^13^C-NMR (CD_3_COCD_3_) δ 14.2, 23.2, 25.5, 29.6, 29.7, 32.3, 34.5, 114.0, 114.9, 116.2, 117.3, 120.3, 124.8, 130.1, 131.5, 141.0, 146.1, 146.5, 152.6, 172.1; HRMS found: 496.08; C_30_H_40_O_6_ requires 496.28.

*3,5-Di-O-palmitoyl piceatannol* (**5d**). Yellow oil, 82% yield. ^1^H-NMR (CDCl_3_+CD_3_OD) δ 0.83 (m, 6H, 2 × C*H*_3_), 1.12-1.38 (m, 48H, 24 × C*H*_2_), 1.66-1.73 (m, 4H, 2 × C*H*_2_), 2.50 (t, *J* = 7.3 Hz, 4H, 2 × C*H*_2_), 6.68-7.01 (m, 8H, C*H_α_*=C*H_β_*, *H*-2,4,6,2’,5’,6’); ^13^C-NMR (CDCl_3_+CD_3_OD) δ 14.0, 22.6, 24.8, 29.1, 29.2, 29.3, 29.4, 29.6, 29.7, 31.9, 34.4, 112.7, 113.7, 115.1, 116.5, 119.8, 124.5, 129.5, 130.6, 140.1, 144.5, 144.8, 151.3, 172.2; HRMS found: 720.21; C_42_H_72_O_6_ requires 720.53.

### 3.4. Acetylation of 3,5-di-O-acetyl piceatannol *(**5a**)*

Compound **5a** (1.0 mmol) was solubilized in pyridine (10 mL) and acetic anhydride was added (10 mL) overnight. At the end of the reaction, a 1 M solution of HCl (10 mL) was added and the final product was extracted with ethyl acetate (3 × 10 mL). The combined organic phases were washed with a solution of NaHCO_3_ (20 mL) and with a saturated solution of NaCl (20 mL), then dried over Na_2_SO_4_. After evaporation of the solvent, and chromatographic purification on silica gel, *3,5,3’,4’-tetra-O-acetyl piceatannol* (**6a**) was isolated in quantitative yield and characterized by spectroscopic data. Colorless oil; ^1^H-NMR (CDCl_3_) δ 2.23 (s, 6H, 2 × C*H*_3_), 2.25 (s, 6H, 2 × C*H*_3_), 6.84 (t, *J* = 2.0 Hz, 1H, *H*-4), 6.96 (d, *J* = 16 Hz, 2H, *CH*=C*H_β_*), 7.02 (d, *J* = 16 Hz, 2H, C*H_α_*=CH), 7.10 (d, *J* = 2.0 Hz, *H*-2,6), 7.18 (d, *J* = 8.5 Hz, *H*-5’), 7.30 (d, *J* = 1.5 Hz, H-2’), 7.34 (dd, *J* = 8.5 and 1.5 Hz, *H*-6’). ^13^C-NMR (CDCl_3_) δ 20.6, 21.1, 114.6, 117.0, 121.2, 123.6, 125. 0, 128.2, 128.8, 139.2, 141.7, 141.7, 142.3, 151.3, 168.2, 168.9; HRMS found: 412.24; C_18_H_16_O_6_ requires 412.12.

### 3.5. Measure of antioxidant activity of compounds ***5a-d***

The radical scavenging activity of compounds **5a-d** was evaluated in the presence of stable radical DPPH• according to the method of Brand-Williams *et al.* [46]. The initial concentration of DPPH• in acetone was controlled for every experiment from a calibration curve made by measuring the absorbance at λ = 517 nm of standard DPPH• solutions at different concentrations. The equation of the curve was ABS_515nm_ = 10,865 × [DPPH•], as determined by linear regression. The compounds **5a-d** were tested at three different concentrations and each concentration was analyzed in triplicate. In a typical procedure, 10, 20 and 30 μL of freshly prepared acetone solution of compounds **5a-d** were added to 2 mL of freshly prepared 184 μM acetone solution of DPPH•. The solutions were shaken for 30 min in the dark at 25 °C. The results were plotted as the percentage of reacted DPPH• [(ABS*_0h_* – ABS*_30min_*)/ABS*_0h_* × 100] vs the concentration (μM) of the added compounds **5a-d**. The results were expressed as SC_50_ that is the concentration of compound required to quench 50% of the initial DPPH• radicals under the experimental conditions given.

## 4. Conclusions

In summary, we have described a simple and efficient procedure to prepare new piceatannol derivatives from naturally occurring resveratrol. These compounds possess a free-catecholic moiety in the B ring and long alkyl chains on the A ring. With these chemical features they exhibited an antioxidant activity similar to that of piceatannol, but a greater lipophilicity, therefore, they may be advatageously used as food antioxidants in bulk lipids or emulsions, and could be further evaluated for possible applications in the pharmaceutical, nutritional and cosmetic fields.

## Figures and Tables

**Table 1:** Aromatic hydroxylation of 3,5-di-*O*-acetyl resveratrol (**4a**) with IBX/Na_2_S_2_O_4_ procedure.

Entry	Solvent	T (°C)	Time (h)	Conv. (%)	Yield of 5a (%)
1	Methanol	0	1	20	20
2	Methanol/Chloroform=1/1	0	1	70	68
3	Dimethyl carbonate	25	2	50	46
4	Dimethyl sulfoxide	25	1	>98	95

**Table 2:** Aromatic hydroxylation of resveratrol derivatives **4a**-**d** with IBX/Na_2_S_2_O_4_ procedure. ^a^

Entry	Substrate	Time (h)	Conv. (%)	Yield (%)
1	**4b**	1	92	**5b**: 88
2	**4c**	2	90	**5c**: 84
3	**4d**	2	90	**5d**: 82

^a^ Oxidation reactions were performed in DMSO at room temperature.

**Table 3:** DPPH• scavenging activity of compounds **5a-d** expressed as SC_50_.

Compound	SC_50_ (μM)^a^ ± SD
**5a**	35.6 ± 2.1
**5b**	32.2 ± 2.5
**5c**	30.0 ± 1.6
**5d**	28.9 ± 1.4

^a^ Each reported value is the mean of three separate measurements.
